# Characterization of Colonizing *Staphylococcus aureus* Isolated from Surgical Wards' Patients in a Nigerian University Hospital

**DOI:** 10.1371/journal.pone.0068721

**Published:** 2013-07-23

**Authors:** Deboye O. Kolawole, Adeniran Adeyanju, Frieder Schaumburg, Akinyele L. Akinyoola, Oladejo O. Lawal, Yemisi B. Amusa, Robin Köck, Karsten Becker

**Affiliations:** 1 Department of Microbiology, Obafemi Awolowo University, Ile-Ife, Nigeria; 2 Department of Surgery, Obafemi Awolowo University, Ile-Ife, Nigeria; 3 Department of Orthopedic Surgery and Traumatology, Obafemi Awolowo University, Ile-Ife, Nigeria; 4 Institute of Public Health, Obafemi Awolowo University, Ile-Ife, Nigeria; 5 Institute of Hygiene, University Hospital Münster, Münster, Germany; 6 Institute of Medical Microbiology, University Hospital Münster, Münster, Germany; Rockefeller University, United States of America

## Abstract

In contrast to developed countries, only limited data on the prevalence, resistance and clonal structure of *Staphylococcus aureus* are available for African countries. Since *S. aureus* carriage is a risk factor for postoperative wound infection, patients who had been hospitalized in surgical wards in a Nigerian University Teaching Hospital were screened for *S. aureus* carriage. All *S. aureus* isolates were genotyped (*spa, agr*) and assigned to multilocus sequence types (MLST). Species affiliation, methicillin-resistance, and the possession of pyrogenic toxin superantigens (PTSAg), exfoliative toxins (ETs) and Panton-Valentine Leukocidin (PVL) were analyzed. Of 192 patients screened, the *S. aureus* carrier rate was 31.8 % (n = 61). Of these isolates, 7 (11.5%) were methicillin-resistant (MRSA). The isolates comprised 24 *spa* types. The most frequent *spa* types were t064, t084, t311, and t1931, while the most prevalent MLST clonal complexes were CC5 and CC15. The most frequent PTSAg genes detected were *seg/sei* (41.0%) followed by *seb* (29.5%), *sea* (19.7%), *seh* (14.7%) and *sec* (11.5). The difference between the possession of classical and newly described PTSAg genes was not significant (63.9% versus 59.0% respectively; *P* = 0.602). PVL encoding genes were found in 39.3% isolates. All MRSA isolates were PVL negative, SCC*mec* types I and VI in MLST CC 5 and CC 30, respectively. Typing of the accessory gene regulator (*agr*) showed the following distribution: *agr* group 1 (n = 20), group II (n = 17), group III (n = 14) and group IV (n = 10). Compared to European data, enterotoxin gene *seb* and PVL-encoding genes were more prevalent in Nigerian methicillin-susceptible *S. aureus* isolates, which may therefore act as potential reservoir for PVL and PTSAg genes.

## Introduction

*Staphylococcus aureus* is a widely recognized human pathogen that continues to represent a significant public health challenge globally. Owing to its broad spectrum of inherent virulence factors, it has been shown to cause a variety of infectious diseases such as superficial skin infections, endocarditis, septicemia, and toxin-mediated diseases, which are often difficult to treat [1], [2]. In addition to its known virulence properties, the ability of methicillin-resistant *S. aureus* strains (MRSA) to consistently evolve resistance mechanisms has led to the emergence of hospital-adapted multi-resistant clones, which became a major cause of nosocomial infections world-wide [3], [4], [5]. The occurrence of MRSA strains in the community (community-associated MRSA) and in livestock (livestock-associated MRSA), as well as the potential risk of import of such strains into the hospital, are also matters of concern [6], [7]. While some data are available on the occurrence of *S. aureus* implicated in various infections [1], [4], [5], [6], [8], [9], [10], [13], [14], [15] there are only few reports describing *S. aureus* colonization pattern in patients from sub-Saharan Africa [11], [12], [13]. In particular, for surgical patients who are predisposed to develop a variety of post-operative events, including post-surgical toxic shock syndrome, data concerning the toxigenic equipment, MRSA prevalence, and clonal structure of *S. aureus* are missing in Africa. It is well known that antibiotic-resistance, toxigenic equipment carriage and clonal analyses in *S. aureus* can provide useful insights into the virulence potential and nature of *S. aureus* populations [1], [2], [16], [17], [18], [19]. Among the several exotoxins secreted by *S. aureus*, Panton-Valentine Leukocidin (PVL), a bi-component cytotoxin encoded by *luk*S-PV and *luk*F-PV genes of the PVL-encoding operon, has been observed in less than 5% of *S. aureus* isolates in Europe and can be associated with necrotic skin lesions including severe necrotizing pneumonia [20], [21]. Moreover, the expression of most *S. aureus* virulence factors is known to be controlled by a complex regulatory network including the accessory gene regulator (*agr*) system which consists of an approximate 3kb genetic locus containing divergent transcription units 20,21,22,23. The *agr* locus is characterized by a polymorphism of its auto-inducing peptide (AIP), and has been shown to reliably divide *S. aureus* into four major *agr* groups. These groups evolved early in staphylococcal evolution [20], [21], [22]. Additionally, molecular typing technologies such as staphylococcal protein A (*spa*) typing and multilocus sequence typing (MLST) provide informative typing results which enable the grouping of individual isolates in clonal lineages [20], [21], [24]. Therefore, the aims of the present study were (i) to prospectively collect *S. aureus* isolates from a hospitalized surgical patient cohort in a Nigerian University Teaching Hospital (ii) to investigate their toxigenic and resistance properties and (iii) to analyze their clonal composition.

## Materials and Methods

### Population

The Obafemi Awolowo University Teaching Hospitals Complex in Ile-Ife has approximately 500 available beds across seven major hospital wards (internal medicine, surgery, orthopedics, psychiatry, gynecology, obstetrics and pediatrics). The hospital serves the city of Ile-Ife in Osun State and is a major referral hospital in Southwestern Nigeria. Exclusion criteria were (i) referral from other hospitals, (ii) superficial skin and soft tissue infections, and (iii) hospitalization for more than 48 hours at the time of sampling. The most common causes of hospitalization on the basis of clinical records were internal fractures (38.8%), cancers (34.6%), peritonitis (8.3%), osteomyelitis (7.2%), and urinary tract infections (5.5%). Demographic and clinical information was collected by interviews, questionnaires and review of clinical records. Institutional Review Board approval was obtained from Obafemi Awolowo University Teaching Hospitals Complex and all patients gave written informed consent to participate in the study.

### Bacterial strains

Between November 2008 and July 2010 all eligible patients hospitalized in the surgical wards (general surgery, pediatric surgery, orthopedics and gynecology) were prospectively screened for *S. aureus* within 48 hours of admittance. Swabs were collected from both anterior nares and from the skin (back of the wrist) using sterile cotton-tipped swab sticks (MicroPoint Diagnostics). A total of 48 strains were isolated from the nares, and 13 strains were recovered from cutaneous specimens. Identification of *S. aureus* and antimicrobial susceptibility testing was performed using VITEK-2 automated systems (BioMérieux, Marcy l'Etoile, France).

### DNA extraction and polymerase chain reaction approaches

DNA extraction was achieved with a QIAamp tissue kit (Qiagen, Hilden, Germany), by following the manufacturer's recommendations. Confirmation of *S. aureus* and methicillin-resistance was achieved by PCR targeting *nuc* and *mec* genes (*mecA* and recently described *mecA*_LGA251_/*mecC*) respectively [25], [26], [27]. Multiplex PCRs for detection of exotoxin genes including exfoliative toxin genes (*eta, etb, etd*), classical staphylococcal PTSAgs (*sea, seb, sec, sed, see* and *tst*), the subsequently described staphylococcal PTSAgs (*seg, seh, sei* and *sej*), and *luk*F-PV and *luk*S-PV genes were conducted as previously described [20], [28], [29].

### *spa* and *agr* typing

To determine the *spa* type of *S. aureus* strains, the polymorphic X-region of the *S. aureus* protein A gene (*spa)* was sequence-typed [18]. Cluster formation of *spa* types was performed by the “based upon repeat patterns” (BURP) algorithm of the StaphType software (Ridom, Münster, Germany) using the default parameters described by Mellmann et al. [19]. Additionally, subtypes of the accessory gene regulator were detected by multiplex PCR as previously described [20], [30]. Multilocus sequence typing (MLST) was performed for *spa* types detected for the first time in this study [24].

### SCC*mec* typing

SCC*mec* typing of MRSA isolates (n = 7) was performed as previously described [31].

### Statistical analysis

Categorical data were compared using the chi-square test. In addition, we report proportions of categorical variables (i.e. virulence factors and antimicrobial resistance). All computations were performed using Epi Info^TM^ 3.5.3 (Centers for Disease Control and Prevention, Atlanta, USA). P values >0.05 were considered not statistically significant.

## Results

### Patient characteristics and *S. aureus* carriage

Among 192 persons tested, the overall prevalence of *S. aureus* carriage was 31.8% (n = 61) with 27.1% (n = 28) among males and 37.1 % (n = 33) among females (*P* = 0.105) (Table 1). The mean age was 39.7 years among carriers of methicillin-susceptible *S. aureus* (MSSA), 31.4 years among MRSA carriers and 44.2 years among *S. aureus* non-carriers (*P* = 0.30). All *S. aureus* were tested *nuc* positive. Seven of the 192 patients (3.7%) were colonized by MRSA and 11.5% of all *S. aureus* were MRSA. The seven MRSA were recovered within the period of sampling from seven patients variously hospitalized in the orthopedic (n = 2), general surgery (n = 4) and pediatric surgery (n = 1) wards. All MRSA isolates were tested positive for *mecA* and negative for *mecC.* Of all 61 *S. aureus* isolates, 73.7% were resistant to penicillin, 42.6% to tetracycline, 3.3% to clindamycin, 16.4% to gentamicin, 9.8% to levofloxacin and 44.2% to trimethoprim/sulfamethoxazole, respectively. Vancomycin resistance was not found.

**Table 1:** Characteristics of the surgical patient cohort studied.

Characteristic^1^	Males	Females	Total
**Population size, ** ***S. aureus*** ** carriage and age**			
Number of participants	103 (53.6)	89 (46.3)	192 (100)
*S. aureus* carriage	28 (27.1)	33 (37.1)	61 (31.8)
Age, years, median (range)	36 (2–86)	41 (1–76)	38 (2–86)
**Duration of hospitalization**			
(≤3 days)	13 (12.6)	22 (24.7)	33 (17.1)
(7 days)	33 (32.0)	30 (33.7)	63 (32.8)
(≥14 days)	57 (55.3)	37 (41.5)	94 (49.0)
**Risk factor for ** ***S. aureus*** ** colonization**			
Recent hospitalization (<6 months)	7 (6.8)	12 (13.4)	17 (8.8)
Recent surgery	3 (2.9)	5 (5.6)	8 (4.1)
Diabetes	4 (3.8)	3 (3.3)	7 (3.6)
Urinary tract catheter	20 (19.4)	12 (13.4)	32 (16.6)
Chest drain	0	2 (2.24)	2 (1.04)
Intravenous fluid (IVF) administration	27 (26.2)	30 (33.7)	57 (29.6)
Pre-surgical patient	48 (46.6)	75 (84.2)	123 (64.0)
Post-surgical patient	55 (53.3)	14 (15.7)	69 (35.9)

1 Data are no.(%) of participants unless otherwise indicated.

### Exotoxin gene detection

Overall, 70.5% (n = 43) of all isolates tested were PTSAg gene-positive. We did not observe significant differences between the prevalence of classical PTSAg genes and the subsequently described enterotoxin and enterotoxin-like genes tested (63.9% vs 59.0% repspectively; *P* = 0.602). Regarding the classical enterotoxin genes, *seb* gene was the most prevalent followed by *sea* gene and *sec* gene. The *sed* gene was detected in two isolates, while *see* gene was not observed. Altogether, three isolates harbored the *tst* gene. The *seg-sei* genes occurred most frequently and strictly in combination with one another while the *seh* gene was observed in nine isolates (Table 2). Several enterotoxin gene combinations were observed including isolates with a combination of two (n = 14, 22.9%), three (n = 20, 32.7%), four (n = 2, 3.6%) and five (n = 1, 1.6%) different genes. The most frequent combination of genes detected was *seg – sei* – *seb* genes occurring in 19.6% (n = 12) of all isolates. In contrast, seven isolates (11.4%) harbored a single PTSAg gene. Overall, three (4.9%) of the isolates were positive for the exfoliative toxin genes; *etb gene* was not detected. While 99% of isolates tested PCR positive to γ–hemolysin encoding genes, the *luk*F-PV and *luk*S-PV genes of the PVL operon were detected in 24 isolates (39%), all of which were MSSA strains. Moreover, 75.9% (n = 41) of the MSSA population were PTSAg gene positive (Table 3): 13% were positive for the classical PTSAg genes (n = 7); 13% exclusively encoded the newly described PTSAg genes (n = 7) while 50% of the MSSA population encoded both classical and newly described PTSAg genes. The *seb* gene was the most frequently observed classical PTSAg gene in the MSSA group (n = 17, 31.4%) while *seg-sei* genes occupied this position (n = 24, 44.4%) among the subsequently described PTSAg genes. The percentage of exfoliative toxin gene-positive MSSA strains was 5.5% (n = 3).

**Table 2:** Results of testing 61 *S. aureus* isolates for staphylococcal PTSAg and ET genes by multiplex PCR.

Positive result of multiplex PCR testing^1^	Nasal (n = 48)		Skin (n = 13)		Total (n = 61)	
	n	%	n	%	n	%
*sea*	7	14.5	5	38.4	12	19.7
*seb*	12	25.0	6	46.1	18	29.5
*sec*	7	14.5	0	0.0	7	11.5
*sed*	2	4.1	0	0.0	2	3.2
*see*	0	0.0	0	0.0	0	0.0
*tst*	2	4.1	1	7.7	3	4.9
*seg-sei*	20	41.6	6	12.5	26	42.6
*seh*	5	10.4	4	30.7	9	14.6
*sej*	1	2.1	0	0.0	1	1.6
*eta*	2	4.1	0	0.0	2	3.2
*etb*	0	0.0	0	0.0	0	0.0
*etd*	1	2.1	0	0.0	1	1.6
*luk*S-PVand *luk*F-PV	20	41.6	5	38.4	25	41.0
*hlg*	48	100.0	12	92.3	60	99.0

1 *sea*, staphylococcal enterotoxin A gene; *seb*, staphylococcal enterotoxin B gene; *sec*, staphylococcal enterotoxin C gene; *sed*, staphylococcal enterotoxin D gene; *see*, staphylococcal enterotoxin E gene; *tst*, toxin shock toxin gene; *seg-sei*, staphylococcal enterotoxin G and staphylococcal enterotoxin I genes; *seh*, staphylococcal enterotoxin H gene; *sej*, staphylococcal enterotoxin J gene; *eta*, exfoliative toxin A gene; *etb*, exfoliative toxin B gene; *etd*, exfoliative toxin D gene; *luk*S-PV and *luk*F-PV, Panton-Valentine leukocidin genes; *hlg*, gamma-hemolysin gene.

**Table 3:** Characteristics of nasal and cutaneous *S. aureus* isolates.

*spa* 1		MLST^2^		Source for MLST classification	PVL^3^	PTSAg/ET gene profile^4^	*agr* 5	Methicillin resistance^6^	SCC*mec* 7	Source	
Type (n)	*spa*-CC	CC	ST							Nasal	Skin
t159 (3)	singleton	CC121	ST121	39, 41	Pos.	*seb, seg, sei*	IV	MSSA	ND	2	1
t2304 (6)	excluded	CC121	ST121	41	Pos.	*seb, seg, sei*	IV	MSSA	ND	3	3
t2304 (1)	excluded	CC 121	ST 121	41	Pos.	*sed, seg, sei*	IV	MSSA	ND	1	–
t084 (2)	spaCC084/2216	CC15	ST15	39, 41	Neg.	*–*	II	MSSA	ND	2	–
t084( 2)	spaCC084/2216	CC15	ST15	39, 41	Neg.	*sec*	II	MSSA	ND	2	–
t084 (1)	spaCC084/2216	CC15	ST15	39, 41	Neg.	*eta*	II	MSSA	ND	1	–
t2216 (2)	spaCC084/2216	CC 15	ST15	39, 41	Neg.	*–*	I, II	MSSA	ND	2	–
t2216 (1)	spaCC084/2217	CC15	ST15	39, 41	Neg.	*eta*	II	MSSA	ND	1	–
t127(1)	spaCC127/321	CC 15	ST1	39, 41	Pos.	*sea, seh*	III	MSSA	ND	1	–
t1931(6)	spaCC1931/7835	CC15	ST1	39	Neg.	*sea, seh*	III	MSSA	ND	3	3
t7835 (1)	spaCC1931/7835	CC15	ST1	This study	Pos.	*tst, seh*	III	MSSA	ND	1	–
t321(1)	spaCC127/321	CC15	ST1	41	Neg.	*tst, sea, seh*	III	MSSA	ND	0	1
t3772 (1)	singleton	CC15	ST25	39, 41	Neg.	*sec, seg, sei/etd*	I	MSSA	ND	1	–
t458 (1)	excluded	CC15	ST97	41	Neg.	*–*	I	MSSA	ND	1	–
t3086 (1)	singleton	CC15	ST188	37	Pos.	*–*	I	MSSA	ND	1	–
t7834 (1)	singleton	CC15	ST2355	This study	Neg.	*sec, seg, sei*	II	MSSA	ND	1	–
t355 (4)	singleton	CC152	ST152	39, 41	Pos.	*–*	I	MSSA	ND	3	1
t8037 (1)	singleton	CC152	ST152	This study	Pos.	*–*	I	MSSA	ND	1	–
t318 (2)	singleton	CC30	ST30	41	Neg.	*seg, sei*	III	MSSA	ND	1	1
t007 (1)	singleton	CC30	ST39	45	Neg.	*–*	III	MRSA	VI	1	–
t095 (1)	singleton	CC45	ST45	34	Neg.	*tst, sec, seg, sei*	I	MSSA	ND	1	–
t002 (1)	spaCC002/311	CC5	ST5	39, 41	Neg.	*seb, sed, seg, sei, sej*	II	MRSA	NT	1	–
t1842(1)	singleton	CC5	ST5	39, 41	Neg.	*sea*	I	MRSA	NT	1	–
t311(1)	spaCC002/311	CC5	ST5	39, 41	Neg.	*seb, seg, sei*	II	MSSA	ND	1	–
t311(2)	spaCC002/311	CC5	ST5	39, 41	Pos.	*sea, seg, sei*	II	MSSA	ND	2	–
t311 (2)	spaCC002/311	CC5	ST5	39, 41	Neg.	*seg, sei*	II	MSSA	ND	2	–
t688(2)	singleton	CC5	ST5	46	Pos.	*seb, seg, sei*	II	MSSA	ND	1	1
t688(1)	singleton	CC5	ST5	46	Pos.	*seg, sei*	II	MSSA	ND	1	–
t064 (1)	singleton	CC5	ST8	36	Neg.	*seb, seg, sei*	I	MSSA	ND	1	–
t064(3)	singleton	CC5	ST8	36	Neg.	*sea, seb*	I	MSSA	ND	2	1
t064(2)	singleton	CC5	ST8	36	Neg.	*seb*	I	MSSA	ND	2	–
t8034 (1)	singleton	CC5	ST72	This study	Neg.	*sec, seg, sei*	I	MSSA	ND	1	–
t037(1)	singleton	CC5	ST241	41	Neg.	*–*	I	MRSA	I	1	–
t194(1)	singleton	CC5	ST250	47	Neg.	*–*	I	MRSA	NT	1	–
t729(2)	singleton	CC88	ST88	39	Neg.	*–*	III	MRSA	NT	2	–

1 *spa*, staphylococcal protein A gene; *spa*CC, *spa* clonal complex inferred by BURP analysis; n, indicates number of isolates with similar identity for all characteristics tested;

2 MLST, multilocus sequence typing; CC, clonal complex; ST, sequence type;

3 PVL, Panton-Valentine leukocidin; pos., positive; neg., negative;

4 PTSAg/ET gene profile, pyrogenic toxin superantigen gene/exfoliative toxin gene profile; -, no PTSAg/ET gene detected;

5 *agr*, accessory gene regulator type;

6 MSSA, methicillin-susceptible *S. aureus*; MRSA, methicillin-resistant *S. aureus*;

7 ND, not done; NT, not type able.

Regarding the MRSA subset, two isolates were PTSAg gene positive (28.5%). One of the MRSA isolates was associated with five PTSAg genes, namely: *seb*, *sed-sej* and *seg-sei* genes. Meanwhile the second MRSA isolate harbored the *sea* gene alone. Exfoliative toxin and PVL encoding genes were not observed in any of the MRSA isolates tested.

### *spa* typing

All 61 *S. aureus* isolates were *spa*-typeable (Table 3), and were associated with distinct *spa* types. Four hitherto undescribed *spa-*types were found, designated t7834, t7835, t8034 and t8037. Among 54 MSSA isolates, we detected 20 s*pa* types (Table 3): the most frequently observed *spa* types were t064, t084, t311, t1931 and t2304, accounting for 54% of all isolates tested. Seven MRSA were associated with 6 *spa* types (t002, t007, t037, t194, t729 and t1842).

The assignment of isolates to clonal lineages by applying *spa* and MLST mappings (http://spa.ridom.de/mlst.shtml), in combination with a literature search and our own MLST analysis revealed that among MSSA isolates, CC15 (ST1, ST15, ST188, ST2355, ST25 and ST97), CC121 (ST121), and CC 5 (ST5, ST8 and ST72) were the most prevalent (Table 3). The MRSA isolates were associated with MLST CC5 (ST5, ST250 and ST2419) CC30 (ST39) and CC88 (ST88). Additionally, BURP clustering of *spa* types resulted in 5 *spa* CCs, 30 Singletons and two excluded *spa* types (t458 and t2304) (Table 3).

### SCC*mec* typing

Two distinct *SCCmec* types (I and VI) were observed among the seven MRSA isolates. However, five of the seven MRSA isolates were not typeable (NT, Table 3).

### *agr* typing

Typing of the accessory gene regulator (*agr*), revealed the following distribution: *agr* group I (n = 20, 32.7%), *agr* group II (n = 17, 27.8%), *agr* group III (n = 14, 22.9%) and *agr* group IV (n = 10, 16.4%). We observed 78% of *seb* gene-positive isolates in two *agr* groups, *agr* group I (n = 6, 33.3%) and *agr* group IV (n = 8, 44.4%). However, *seg-sei* gene-positive isolates dominated in *agr* group IV (n = 10, 40%) and *agr* group II (n = 10; 40%). All *seh* gene-positive isolates belonged to *agr* group III (n = 9) and (MLST) ST1. Similarly, *S. aureus* isolates that tested positive for *tst* gene were observed in *agr* group III. Exfoliative toxin gene-positive isolates observed in this study belonged to *agr* group I and *agr* group II. Moreover, PVL genes were mostly associated with *agr* group IV (44%) and (MLST) ST121 while being least associated with *agr* group III (8%). Furthermore, *agr* grouping of MRSA strains revealed three MRSA strains in *agr* group I (42.8%) and *agr* group III (42.8%) while one MRSA strain (14.2%) belonged to *agr* group II. In contrast, MSSA strains more frequently belonged to *agr* group I (n = 17, 31.4%) and *agr* group II (n = 16, 29.6%) than *agr* group III (n = 11, 20.3%) and *agr* group IV (n = 10,18.5%).

## Discussion

We analyzed the clonal structure, toxin gene equipment and resistance pattern of 61*S. aureus* carriage isolates obtained from a surgical patient cohort (n = 192) in Nigeria. While 3.9% of the patients in the cohort studied were colonized by MRSA, Heysell et al. [11] reported on a high prevalence of multidrug-resistant MRSA nasal carriage (29%) in a district hospital in South Africa. Interestingly, in epidemiological studies involving persons on the Cape Verde islands as well as Central African Babongo Pygmies, no MRSA isolate was found [12], [32]. However, in studies characterizing infection-related *S. aureus* isolates, higher and increasing MRSA rates (11.1–47.4%) have been reported in the North and sub-Saharan African region [9], [13], [33]. Reports on the prevalence of PTSAg genes and the detection of respective toxin genes differ greatly depending on the geographic affiliation, the population structure tested, and the range of staphylococcal PTSAg genes included. In particular, the recent detection of enterotoxins and enterotoxin gene-like toxin genes beyond the classical spectrum (such as enterotoxins SEG – SEJ, SEK – SER and SEU) has increased the percentage of those *S. aureus* isolates bearing at least one of the PTSAg encoding genes [28], [34]. Moreover, like SEC (staphylococcal enterotoxin C), several of the newly described enterotoxins are polymorphic, being characterized by the occurrence of nucleotide variants, additionally challenging their precise detection [29]. In this study, 11.4% of the *S. aureus* isolates were shown to harbor at least one of the PTSAg gene family members. This is in accordance with other studies of different geographic origin [23], [28], [35]. However, these studies differ from our investigation because they included *S. aureus* isolates obtained from the clinical care setting. It is well documented that the smallest amounts of staphylococcal enterotoxins may induce T-cell stimulation resulting in systemic illness such as staphylococcal enterotoxin-induced shock and autoimmunity [28], [36]. Given the detection of a significant amount of toxin genes including *tst* gene in the present study, post-operative patients hospitalized in the surgical wards may be at risk of post-surgical toxic shock syndrome [16], [17]. For SEH, emetic activity has been shown and this PTSAg is considered a potential causative agent for food poisoning [37]. Importantly, a high percentage of *seh* gene-positive isolates (14.7%) was found in our study relative to a low prevalence reported for this enterotoxin gene in other studies investigating non-food borne-related *S. aureus* isolates: 5.4% [28], 4% [35], and 4.3% [38]. Peck et al. [39] reported significant differences in the prevalence of selected enterotoxin genes, including *seh* gene in *S. aureus* isolates obtained from blood compared to nasal isolates (7.2% vs. 30.5%). However for food borne isolates, higher percentages of *seh* gene-positive isolates have been observed. Aydin et al. [40] reported on 16.3% *seh* gene-positive *S. aureus* isolates obtained from food samples, particularly from meat.

We have found a high prevalence (44%) of *luk*S-PV and *luk*F-PV genes of the PVL operon in MSSA carriage isolates. For *S. aureus* isolates obtained from wound infection, similar results have been reported [15], [37]. Similarly, Schaumburg et al. [13] reported on Gabonese *S. aureus* isolates from both, asymptomatic carriers and infections with high percentages of PVL-positive MSSA isolates. Moreover, for a remote Central African population with limited access to health care facilities, a high prevalence of PVL-positive MSSA was also detected [12]. Since PVL-encoding genes are carried on prophages as mobile genetic elements, which enable incorporation into *S. aureus* lineages via horizontal transfer, the Central and West African region may act as a potential major reservoir for the PVL virulence factor with considerable impact on regional as well as global health care systems. Molecular typing of the MRSA carriage isolates characterized in this study revealed that they belonged to *spa* types indicative for the clonal complexes *spa*-CC5 (t002, t037, t194, t1842), *spa*-CC30 (t007) and *spa*-CC88 (t729), all of which have been previously described in African countries [12], [15], [41], [42].

In contrast, among MSSA isolates, *spa* types indicative for MLST CC15 (ST1/ST215; mostly *spa* types t084, t1931) and MLST CC5 (ST1/ST8; mostly *spa* types t064, t311) were predominant. While similar findings have been reported from other studies on the molecular structure of African MSSA [10], [43], we did not observe *S. aureus* strains of MLST sequence types ST2250, and ST2277 in the present study. Of note, four hitherto undescribed *spa* types: t7834, t7835, t8034 and t8037, associated with ST2355, ST1, ST72 and ST152 respectively, were detected in this study. Furthermore, a high prevalence of PVL-positive MSSA isolates (16.4%) of *spa* types t159 and t2304 (clonal lineage MLST ST121/CC121), which belonged to *agr* group IV, was observed in our study. These findings are consistent with previous reports from Africa and other regions of the world including Europe and arctic Russia [10], [15], [44].

In summary, we documented a 3.9% prevalence of MRSA colonization among 192 patients screened. Analysis of the toxin gene content of the Nigerian *S. aureus* isolates revealed a relatively high overall prevalence of PTSAg genes. In particular, the *seh* gene encoding for an enterotoxin which can induce emetic disease was found in association with ST1. While no PVL-positive MRSA isolates were detected, PVL-encoding genes were highly frequent among MSSA isolates of different clonal lineages. Since we characterized carriage isolates of *S. aureus* obtained from surgery wards' patients in Nigeria, this study represents an important reference point for understanding the virulence potential and clonal epidemiology of the strains studied. While the role of the accessory gene regulator in *S. aureus* colonization is yet unclear, our study nonetheless provides further insight in to the clonal nature of the strains studied given the well-demonstrated relationship between accessory gene regulator groups and *S. aureus* clonal evolution [20], [22].

## References

[1] Fournier B (2008) Global regulators of *Staphylococcus aureus* virulence genes: In Lindsay JA (ed.) *Staphylococcus* molecular genetics. Caister Academic Press. 137–150.

[2] Becker K, von Eiff C (2011) *Staphylococcus*, *Micrococcus*, and other catalase-positive cocci. In: Versalovic J, Carroll KC, Funke G, Jorgensen JH, Landry HL, Warnock DW, editors. Manual of Clinical Microbiology (10th ed.). ASM press, Washington, D.C.

[3] Deurenberg RH, Vink C, Kalenic S, Friedrich AW, Bruggeman CA, et al (2007) The molecular evolution of methicillin-resistant *Staphylococcus aureus*. Clin.Microbiol.Infect. 13: 222–235. 10.1111/j.1469-0691.2006.01573.x 17391376

[4] Grundmann H, Aanensen DM, van den Wijngaard CC, Spratt BG, Harmsen D, et al (2010) Geographic distribution of *Staphylococcus aureus* causing invasive infections in Europe: a molecular-epidemiological analysis. PLoS Med. 7: e1000215. 10.1371/journal.pmed.1000215 PMC2796391 20084094

[5] Köck R, Becker K, Cookson B, van Gemert-Pijnen JE, Harbarth S, et al.. (2010) Methicillin-resistant *Staphylococcus aureus* (MRSA): burden of disease and control challenges in Europe. Euro Surveill. 15: pii = 19688. 10.2807/ese.15.41.19688-en 20961515

[6] Davis SL, Perri MB, Donabedian SM, Manierski C, Singh A, et al (2007) Epidemiology and outcomes of community-associated methicillin-resistant *Staphylococcus aureus* infection. J.Clin.Microbiol. 45: 1705–1711. 10.1128/JCM.02311-06 PMC1933099 17392441

[7] Köck R, Harlizius J, Bressan N, Laerberg R, Wieler LH, et al (2009) Prevalence and molecular characteristics of methicillin-resistant *Staphylococcus aureus* (MRSA) among pigs on German farms and import of livestock-related MRSA into hospitals. Eur.J.Clin.Microbiol.Infect.Dis. 28: 1375–1382. 10.1007/s10096-009-0795-4 PMC2772956 19701815

[8] Adekeye JD, Adesiyun AA (1984) Frequency of isolation of enterotoxigenic staphylococci from milk of nursing mothers in Kaduna, Nigeria. J.Hyg.(Lond) 93: 531–538. 6512254 10.1017/s0022172400065104 PMC2129477

[9] Antri K, Rouzic N, Dauwalder O, Boubekri I, Bes M, et al (2011) High prevalence of methicillin-resistant *Staphylococcus aureus* clone ST80-IV in hospital and community settings in Algiers. Clin.Microbiol.Infect. 17: 526–532. 10.1111/j.1469-0691.2010.03273.x 20518793

[10] Breurec S, Zriouil SB, Fall C, Boisier P, Brisse S, et al (2011) Epidemiology of methicillin-resistant *Staphylococcus aureus* lineages in five major African towns: emergence and spread of atypical clones. Clin.Microbiol.Infect. 17: 160–165. 10.1111/j.1469-0691.2010.03219.x 20298267

[11] Heysell SK, Shenoi SV, Catterick K, Thomas TA, Friedland G (2011) Prevalence of methicillin-resistant *Staphylococcus aureus* nasal carriage among hospitalised patients with tuberculosis in rural Kwazulu-Natal. S.Afr.Med.J. 101: 332–334. 10.7196/samj.4462 PMC3628595 21837877

[12] Schaumburg F, Köck R, Friedrich AW, Soulanoudjingar S, Ngoa UA, et al (2011) Population structure of *Staphylococcus aureus* from remote African Babongo Pygmies. PLoS Negl.Trop.Dis. 5: e1150. 10.1371/journal.pntd.0001150 PMC3091839 21572985

[13] Schaumburg F, Ngoa UA, Kösters K, Köck R, Adegnika AA, et al (2011) Virulence factors and genotypes of *Staphylococcus aureus* from infection and carriage in Gabon. Clin.Microbiol.Infect. 17: 1507–1513. 10.1111/j.1469-0691.2011.03534.x 21595798

[14] Shittu AO, Lin J, Kolawole DO (2006) Antimicrobial susceptibility patterns of *Staphylococcus aureus* and characterization of MRSA in southwestern Nigeria. WOUNDS 18: 77–84.

[15] Shittu AO, Okon K, Adesida S, Oyedara O, Witte W, et al (2011) Antibiotic resistance and molecular epidemiology of *Staphylococcus aureus* in Nigeria. BMC Microbiol. 11: 92. 10.1186/1471-2180-11-92 PMC3112067 21545717

[16] Lowy FD (1998) *Staphylococcus aureus* infections. N.Engl.J.Med. 339: 520–532. 10.1056/NEJM199808203390806 9709046

[17] Bohach GA, Fast DJ, Nelson RD, Schlievert PM (1990) Staphylococcal and streptococcal pyrogenic toxins involved in toxic shock sydrome and related illnesses. Critical Rev. Microbiol. 17: 251–272. 10.3109/10408419009105728 2206394

[18] Mellmann A, Weniger T, Berssenbrügge C, Keckevoet U, Friedrich AW, et al (2008) Characterization of clonal relatedness among the natural population of *Staphylococcus aureus* strains by using *spa* sequence typing and the BURP (based upon repeat patterns) algorithm. J.Clin.Microbiol. 46: 2805–2808. 10.1128/JCM.00071-08 PMC2519486 18524961

[19] Mellmann A, Weniger T, Berssenbrügge C, Rothganger J, Sammeth M, et al (2007) Based Upon Repeat Pattern (BURP): an algorithm to characterize the long-term evolution of *Staphylococcus aureus* populations based on *spa* polymorphisms. BMC Microbiol. 7: 98. 10.1186/1471-2180-7-98 PMC2148047 17967176

[20] von Eiff C, Friedrich AW, Peters G, Becker K (2004) Prevalence of genes encoding formembers of the staphylococcal leukotoxin family among clinical isolates of *Staphylococcus aureus*. Diagn.Microbiol.Infect.Dis. 49: 157–162. 10.1016/j.diagmicrobio.2004.03.009 15246504

[21] Lina G, Boutite F, Tristan A, Bes M, Etienne J, et al (2003) Bacterial competition for human nasal cavity colonization: role of staphylococcal *agr* alleles. Appl.Environ.Microbiol. 69: 18–23. 10.1128/AEM.69.1.18-23.2003 PMC152380 12513972

[22] Jarraud S, Mougel C, Thioulouse J, Lina G, Meugnier H, et al (2002) Relationships between *Staphylococcus aureus* genetic background, virulence factors, *agr* groups (alleles), and human disease. Infect Immun. 70: 631–641. 10.1128/IAI.70.2.631-641.2002 PMC127674 11796592

[23] Novick RP (2003) Autoinduction and signal transduction in the regulation of staphylococcal virulence. Mol Microbiol 48: 1429–1449. 12791129 10.1046/j.1365-2958.2003.03526.x

[24] Enright MC, Day NP, Davies CE, Peacock SJ, Spratt BG (2000) Multilocus sequence typing for characterization of methicillin-resistant and methicillin-susceptible clones of *Staphylococcus aureus*. J.Clin.Microbiol. 38: 1008–1015. 10.1128/jcm.38.3.1008-1015.2000 PMC86325 10698988

[25] Becker K, Pagnier I, Schuhen B, Wenzelburger F, Friedrich AW, et al (2006) Does nasal cocolonization by methicillin-resistant coagulase-negative staphylococci and methicillin-susceptible *Staphylococcus aureus* strains occur frequently enough to represent a risk of false-positive methicillin-resistant *S. aureus* determinations by molecular methods? J.Clin.Microbiol. 44: 229–231. 10.1128/JCM.44.1.229-231.2006 PMC1351978 16390977

[26] Brakstad OG, Aasbakk K, Maeland JA (1992) Detection of *Staphylococcus aureus* by polymerase chain reaction amplification of the *nuc* gene. J.Clin.Microbiol. 30: 1654–1660. 10.1128/jcm.30.7.1654-1660.1992 PMC265359 1629319

[27] Kriegeskorte A, Ballhausen B, Idelevich EA, Köck R, Friedrich AW, et al (2012) Human MRSA isolates with novel genetic homolog, Germany. Emerg.Infect.Dis. 18: 1016–1018. 10.3201/eid1806.110910 PMC3358142 22608212

[28] Becker K, Friedrich AW, Lubritz G, Weilert M, Peters G, et al (2003) Prevalence of genes encoding pyrogenic toxin superantigens and exfoliative toxins among strains of *Staphylococcus aureus* isolated from blood and nasal specimens. J.Clin.Microbiol. 41: 1434–1439. 10.1128/JCM.41.4.1434-1439.2003 PMC153929 12682126

[29] Blaiotta G, Fusco V, von Eiff C, Villani F, Becker K (2006) Biotyping of enterotoxigenic *Staphylococcus aureus* by enterotoxin gene cluster (*egc*) polymorphism and *spa* typing analyses. Appl.Environ.Microbiol. 72: 6117–6123. 10.1128/AEM.00773-06 PMC1563601 16957237

[30] Lina G, Piémont Y, Godail-Gamot F, Bes M, Peter MO, et al (1999) Involvement of Panton-Valentine leukocidin-producing *Staphylococcus aureus* in primary skin infections and pneumonia. Clin. Infect. Dis. 29: 1128–32. 10.1086/313461 10524952

[31] Kondo Y, Ito T, Ma XX, Watanabe S, Kreiswirth BN, et al (2007) Combination of multiplex PCRs for staphylococcal cassette chromosome *mec* type assignment: rapid identification system for *mec*, *ccr*, and major differences in junkyard regions. Antimicrob. Agents Chemother. 51: 264–274. 10.1128/AAC.00165-06 PMC1797693 17043114

[32] Aires de Sousa M, Santos Sanches I, Ferro ML, de Lencastre H (2000) Epidemiological study of staphylococcal colonization and cross-infection in two West African Hospitals. Microb.Drug Resist. 6: 133–141. 10.1089/107662900419447 10990268

[33] Truong H, Shah SS, Ludmir J, Twananana EO, Bafana M, et al (2011) *Staphylococcus aureus* skin and soft tissue infections at a tertiary hospital in Botswana. S.Afr.Med.J. 101: 413–416. 21920078

[34] Becker K, Friedrich AW, Peters G, von Eiff C (2004) Systematic survey on the prevalence of genes coding for staphylococcal enterotoxins SElM, SElO, and SElN. Mol.Nutr.Food Res. 48: 488–495. 10.1002/mnfr.200400044 15515095

[35] Hu DL, Omoe K, Inoue F, Kasai T, Yasujima M, et al (2008) Comparative prevalence of superantigenic toxin genes in meticillin-resistant and meticillin-susceptible *Staphylococcus aureus* isolates. J.Med.Microbiol. 57: 1106–1112. 10.1099/jmm.0.2008/002790-0 18719180

[36] Miethke T, Wahl C, Regele D, Gaus H, Heeg K, et al (1993) Superantigen mediated shock: a cytokine release syndrome. Immunobiology 189: 270–284. 8125513 10.1016/S0171-2985(11)80362-1

[37] Su YC, Wong ACL (1995) Identification and purification of a new staphylococcal enterotoxin, H. Appl.Environ.Microbiol. 61: 1438–1443. 10.1128/aem.61.4.1438-1443.1995 PMC167401 7747964

[38] El-Huneidi W, Bdour S, Mahasneh A (2006) Detection of enterotoxin genes *seg*, *seh*, *sei*, and *sej* and of a novel *aroA* genotype in Jordanian clinical isolates of *Staphylococcus aureus*. Diagn.Microbiol.Infect.Dis. 56: 127–132. 10.1016/j.diagmicrobio.2006.04.002 16725300

[39] Peck KR, Baek JY, Song JH, Ko KS (2009) Comparison of genotypes and enterotoxin genes between *Staphylococcus aureus* isolates from blood and nasal colonizers in a Korean hospital. J.Korean Med.Sci. 24: 585–591. 10.3346/jkms.2009.24.4.585 PMC2719184 19654937

[40] Aydin A, Sudagidan M, Muratoglu K (2011) Prevalence of staphylococcal enterotoxins, toxin genes and genetic-relatedness of foodborne *Staphylococcus aureus* strains isolated in the Marmara Region of Turkey. Int.J.Food Microbiol. 148: 99–106. 10.1016/j.ijfoodmicro.2011.05.007 21652103

[41] Ghebremedhin B, Olugbosi MO, Raji AM, Layer F, Bakare RA, et al (2009) Emergence of a community-associated methicillin-resistant S*taphylococcus aureus* strain with a unique resistance profile in Southwest Nigeria. J.Clin.Microbiol. 47: 2975–2980. 10.1128/JCM.00648-09 PMC2738091 19571020

[42] Ruimy R, Angebault C, Djossou F, Dupont C, Epelboin L, et al (2010) Are host genetics the predominant determinant of persistent nasal *Staphylococcus aureus* carriage in humans? J.Infect.Dis. 202: 924–934. 10.1086/655901 20677941

[43] Shittu A, Oyedara O, Abegunrin F, Okon K, Raji A, et al (2012) Characterization of methicillin-susceptible and -resistant staphylococci in the clinical setting: a multicentre study in Nigeria. BMC Infect Dis. 12: 286. 10.1186/1471-2334-12-286 PMC3529121 23121720

[44] Zaraket H, Shabana II, Nevzorova V, Turcutyuicov V, Suzuk H (2010) Molecular characterization and susceptibility of methicillin-resistant and methicillin-susceptible *Staphylococcus aureus* isolates from hospitals and the community in Vladivostok, Russia T. Baranovich. Clin.Microbiol.Infect. 16: 575–582. 10.1111/j.1469-0691.2009.02891.x 19681959

[45] European Food Safety Authority (2009) Analysis of the baseline survey on the prevalence of methicillin-resistant *Staphylococcus aureus* (MRSA) in holdings with breeding pigs, in the EU, 2008 - Part A: MRSA prevalence estimates. EFSA J. 7: 1376.

[46] Torres-Sangiao E, Pérez-Castro S, Fernández-Natal MI, Cisterna-Cáncer R, Zapico-González M, et al (2012) Identification of international circulating lineages of meticillin-resistant S*taphylococcus aureus* in the north of Spain and their glycopeptide and linezolid susceptibility. J.Med.Microbiol. 61: 305–307. 10.1099/jmm.0.036889-0 21980043

[47] Cuny C, Friedrich A, Kozytska S, Layer F, Nubel U, et al (2010) Emergence of methicillin-resistant *Staphylococcus aureus* (MRSA) in different animal species. Int.J.Med.Microbiol. 300: 109–117. 10.1016/j.ijmm.2009.11.002 20005777

